# Nutrition and Propagation of Flowers and Trees

**Published:** 1889-11-09

**Authors:** T. G. Headley


### Nutrition and Propagation of Flowers and Trees.

By Rev. T. G. Headley.

The lecturer (Mr. Gardiner) on flowers, at the meeting of the British Association in Newcastle, said it was a very common idea to suppose that there was no battle or struggle for life amongst flowers, and that they neither toil nor spin; but the lecturer said this was an entirely mistaken and erroneous idea, and that, on the contrary, there was an immense struggle for the survival of the fittest.

And, amongst other instances of the struggle going on amongst plants for nutrition and the survival of the fittest, the lecturer related how Brazil nuts (which our children buy at six a penny) grew in a cluster of twenty-five, which were enclosed in a huge shell as big as one's hat, out of which shell there was no possible egress, excepting through a very small hole at the top, through which alone nutrition and light could reach the nuts, and from out of which alone the nut (or nuts) within could obtain egress to grow and reproduce itself. But as the aperture in this huge shell was so small as to make it impossible for more than one nut out of the twenty-five nuts to obtain egress, there was consequently an immense struggle amongst the nuts to reach this aperture for the reproduction and survival of the fittest. And as the lecturer said this struggle for nutrition and propagation was going on in all life, and was the beginning and end of all life, there were many ladies in the audience who thought they ought to have been offended with this remark of the lecturer, and not have attended to hear it.

But when it is believed that man has a soul or spirit independent of the body, then the nutrition and propagation of the soul or spirit becomes as much, and even more, an object of life as the nutrition and propagation of the body.

And the nutrition and propagation of the soul or spirit is (1) the education of the mind in a knowledge and love of what is good and true, and then (2) the propagation of that knowledge and love to the world, so that it may grow and be reproduced again and again, until the whole world is filled with a knowledge and love thereof, and so God becomes all in all.

And thus, when the soul is included as well as the body, the theory of the lecturer that nutrition and propagation is the beginning and end of all life would be accepted by all, and equally as much by the ladies as the gentlemen.

